# Incidence and risk factors for lack of recovery in patients undergoing major abdominal noncardiac surgery in Sweden: a prospective observational study

**DOI:** 10.1016/j.bjao.2026.100645

**Published:** 2026-07-29

**Authors:** Sara Lyckner, Michelle S. Chew, Anna Neu, Helén Didriksson, Carina Jonsson, Henrik Andersson, Jesper Sperber

**Affiliations:** 1 Department of Anaesthesiology and Intensive Care, Biomedical and Clinical Sciences, Linköping University, Linköping, Sweden; 2 Centre for Clinical Research Sörmland, Uppsala University, Eskilstuna, Sweden; 3 Perioperative Medicine and Intensive Care, Karolinska University Hospital Huddinge, Huddinge, Sweden; 4 Division of Anesthesiology and Intensive Care, CLINTEC, Karolinska Institute, Stockholm, Sweden

**Keywords:** MINSS, myocardial injury, patient-reported outcome measures, postoperative complications, postoperative recovery, quality of recovery, QoR-15, risk factors

## Abstract

**Background:**

Patients’ experiences of postoperative recovery provide insights beyond traditional outcomes. Postoperative complications impair recovery, prolong hospital stay, and affect well-being. Long-term data on recovery patterns, particularly regarding complications, are scarce but essential for guiding perioperative care.

**Methods:**

A pre-planned nested cohort study within the Myocardial Injury in Non-Cardiac Surgery in Sweden study was conducted at two Swedish hospitals; 124 adult patients undergoing major noncardiac surgery were followed for up to 1 yr. Lack of recovery was defined as a ≥6-point decrease in Quality of Recovery-15 (QoR-15) score from preoperative baseline. We assessed the incidence and risk factors, described the course of recovery, and examined the association between early lack of recovery and days alive and out of hospital (DAOH) at 30 days after surgery.

**Results:**

Lack of recovery was reported by 58%, 46%, and 24% of patients at 7–10 days, 30 days, and 1 yr, respectively. Complications occurred in 49% and were associated with a two-fold increase in the odds of lack of recovery at 30 days (adjusted odds ratio 2.60 [1.18–5.73]; *P*=0.018), independent of age, ASA physical classification score, and sex. Patients with complications reported lower QoR-15 scores and a slower recovery over time. Early lack of recovery was associated with 3.7 fewer days alive and out of hospital (mean difference −3.69 [−6.54 to −0.83]; *P*=0.012).

**Conclusions:**

Half of the study group reported a lack of recovery at 1 week, with persistence at 1 month. Complications were common and strongly associated with lack of recovery. Although recovery is slower in patients with complications, most regain or exceed baseline levels by 1 year. Early lack of recovery was linked to reduced days alive and out of hospital.

**Editor’s key points**

• In this Swedish database, 58% of patients experienced a clinical lack of recovery at 1 week after surgery, with nearly half still failing to reach baseline health at 1 month.
• Postoperative complications were the primary driver of poor outcomes; independently associated with a more than a two-fold increase in the odds of failing to recover by 30 days.
• Patients reporting an early lack of recovery at 1 week had an average of 3.7 fewer days alive and out of hospital, demonstrating that patient-reported metrics can predict overall healthcare burden.
• Although most patients eventually regained their baseline status by 1 yr, approximately one in four patients still reported a persistent lack of recovery 12 months after surgery.
• Because age and baseline comorbidities did not significantly predict recovery in this cohort, the findings suggest that preventing perioperative complications is the most critical factor for improving long-term patient well-being.

Recovery after major surgery is a complex process that is influenced by multiple patient and surgical factors. For many patients, the recovery process after surgery continues well beyond hospital discharge. Factors such as longer and more invasive surgical procedures, pain, advanced age, female sex, and comorbidities such as diabetes mellitus and hypertension have been implicated in affecting recovery. However, their relative contributions to sustained, delayed, or incomplete recovery are not well established.^1^^,^^2^

Although traditional clinical outcomes such as death, morbidity, cancer recurrence, and length of hospital stay remain essential indicators of surgical safety and effectiveness, they provide a limited description of the patient’s postoperative experiences. These outcomes primarily capture outcomes from a clinical or medical perspective and may fail to reflect how patients feel, function, and recover after surgery. Patients frequently describe recovery in broader terms, including the ability to return to daily activities and regain a sense of well-being.^3^^,^^4^

Patient-reported outcome measures (PROMs), such as Quality of Recovery (QoR), are key tools for assessing postoperative recovery from patients’ perspectives.^5^, ^6^, ^7^ The QoR-15 is a composite questionnaire covering five domains: physical comfort, physical independence, emotional state, psychological support, and pain.^8^ Definitions of poor postoperative recovery vary. One measure is the minimal clinically important difference (MCID), defined as a six-point change in QoR-15 score.^9^

Postoperative recovery can be understood as a dynamic process unfolding over time. When assessed repeatedly, recovery can be conceptualised as a course that captures patient-relevant changes rather than isolated postoperative states. Previous studies in minor surgery indicate that approximately 30% of patients experience poor postoperative recovery as measured by QoR-15.^10^^,^^11^ Patients undergoing more extensive surgery are at even higher risk.^12^ Large-scale epidemiological studies show that approximately one in five surgical patients will develop postoperative complications.^13^^,^^14^ These complications are associated with longer hospital stay, impaired recovery, disability, and reduced quality of life.^15^, ^16^, ^17^, ^18^, ^19^

Postoperative recovery often relies on patient-reported outcomes (PROs), as the QoR-15 is not universally adopted and can be difficult to implement. Alternative measures such as days alive and out of hospital (DAOH) reflect survival, hospital stay, complications, and healthcare utilisation.^20^ Although 30-day DAOH has been associated with increased perioperative risk and prolonged hospitalisation,^21^^,^^22^ it is unclear whether early lack of recovery reduces DAOH.

Evidence regarding risk factors for poor postoperative recovery and long-term recovery courses after major surgery remains limited and inconclusive.^23^ Addressing these gaps is essential to identify early signals of delayed recovery and to improve patient-centred perioperative care.

## Aim

The primary aims were to investigate the incidence and risk factors for lack of recovery after major noncardiac surgery and to describe patient-reported course of recovery over 1 year. The secondary aim was to assess whether early lack of recovery at 1 week was associated with DAOH at 30 days.

## Methods

### Study design and setting

The study was a pre-planned nested cohort within the Myocardial Injury in Non-Cardiac Surgery in Sweden (MINSS) study (NCT 03436238), consisting of a convenience sample of 124 patients recruited from two hospitals in Sweden, Eskilstuna Hospital and Linköping University Hospital. The study was approved by the Regional Ethical Review Committee in Linköping, Sweden (Dnr: 2017/23-312). The MINSS study was a prospective, observational, assessor-blinded study investigating cardiac biomarkers and outcomes in patients undergoing major noncardiac surgery. The inclusion criteria were age 50 years or older, general anaesthesia, hospital overnight stay, and written consent. For this sub-study, understanding written and spoken was an additional criterion. Major noncardiac surgery was defined as major or complex major, according to the Surgical Outcome Risk Tool.^24^ The exclusion criteria for the MINSS study were acute coronary syndromes, new or decompensated heart failure at presentation, severe aortic stenosis, severely reduced ejection fraction (<40%), and inability to follow the procedures of the study or lack of capacity to give informed consent. Transplantation, trauma, endocrine, and vascular surgeries were excluded owing to their specific risk profiles. This study is reported in accordance with the Strengthening the Reporting of Observational Studies in Epidemiology reporting guidelines (Supplementary Table 1).

### Study procedures

The QoR-15 questionnaire includes 15 items, each scored 0–10, yielding a total score ranging from 0 to 150, with higher scores indicating better recovery. QoR-15 data were collected preoperatively (baseline), at 7–10 days after surgery, primarily in the hospital and, via telephone. Follow-up assessments were also conducted at 30 days and 1 year after surgery. Patients not reached at 7–10 days were followed up at later time points. All assessments were conducted by trained study nurses.

Patient characteristics, complications, length of surgery, hospital stay, and mortality up to 1 year after surgery were extracted from the MINSS study database. Complications registered in the study included perioperative myocardial injury (PMI); major adverse cardiac and cerebrovascular events defined as acute myocardial infarction, heart failure, new arrhythmia, nonfatal cardiac arrest, angina, pulmonary oedema, and stroke; delirium; infections (superficial and deep wound infections, bloodstream infections, urinary tract infection, pneumonia); pulmonary embolism; anastomotic leakage; postoperative bleeding; and acute kidney injury. Complications within 30 days were recorded from clinical records as present or absent, without severity grading.

### Outcomes

The primary outcomes were lack of recovery, defined as a postoperative decrease in QoR-15 corresponding to the MCID of ≥6 points,^9^ compared from baseline and assessed at 7–10 days, 30 days, and 1 yr after surgery, and the course of QoR-15 scores up to 1 yr after surgery. The secondary outcome was DAOH at 30 days. DAOH was calculated as days after hospital discharge up to 30 days, and the one patient who died was assigned zero days.

### Sample size

This study was based on a convenience sample of 124 patients. Given the observational design, no formal *a priori* sample calculation was performed. Based on previous studies, 30% of patients were expected to meet the criterion for lack of recovery.^10^^,^^11^ Given this event rate, the sample size would allow adjustment for three to four variables in multivariable analyses. A *post hoc* power analysis was performed based on the observed effect sizes and event rates.

### Statistical analysis

Data distribution was assessed using the Kolmogorov–Smirnov test and, as appropriate, presented as mean and standard deviation, median and interquartile range (IQR), or frequencies (%). To examine factors associated with lack of recovery at 30 days and 1 year, separate binary logistic regression models were performed. The outcome for the multivariable model was lack of recovery. Independent variables in the model (complications, age, ASA class, and sex) were selected based on clinical plausibility and previous studies.^1^^,^^2^

The course of recovery was analysed using a linear mixed-effects model, with QoR-15 scores as the outcome. Time and relevant risk factors were fixed effects of patients with and without complications.

We assessed the association between lack of recovery on DAOH using multivariable linear regression, with lack of recovery, age, ASA class, and sex as independent variables. The distribution of DAOH was assessed for potential bimodality using visual inspection of histograms and density plots. No major violations of linearity or homoscedasticity were seen.

If individual items in the 15-item questionnaire were missing, they were replaced with the group mean for that item. The proportion of missing data across all items was <1%.

Individuals lost to follow-up at 1 year were analysed regarding patient characteristics and QoR-15 data. No systematic differences were seen.

All analyses were conducted using IBM SPSS software, version 29.0 (IBM SPSS Statistics IBM Corporation, Armonk, NY, USA), and a two-sided *P*-value of <0.05 was considered statistically significant.

### Data availability

The datasets generated and analysed during the current study are available from the corresponding author on reasonable request.

## Results

**Fig 1:**
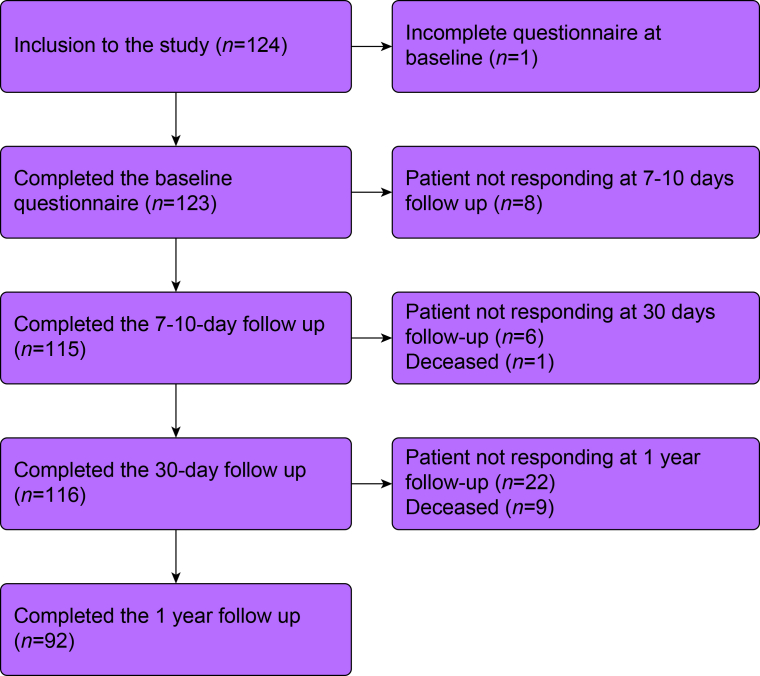
Study flowchart describing inclusion and the Quality of Recovery-15 follow-up.

The MINSS study at the two hospitals consisted of 891 patients; of these, 124 patients were enrolled in this sub-study. Most patients completed all follow-up assessments; fewer than 25% were lost within 1 year. At 30 days, one patient was deceased, and at 1 year, 10 patients (8%) were deceased (Fig. 1). The mean age was 70 year (9), and the most common ASA class was II (83, 66%). Lack of recovery was reported by 58%, 46%, and 24% of patients at 7–10 days, 30 days, and 1 year after surgery, respectively. Further details regarding patient characteristics and QoR-15 values are presented in Table 1.

**Table 1:** Characteristics of the whole study group and the subgroups with and without complications at 30 days. IQR, interquartile range; LoR, lack of recovery; m, median; QoR-15, Quality of Recovery-15; SD, standard deviation.

The study group characteristics		Whole population (*n*=124)	Complications (*n*=61)	No complications (*n*=63)
**Age, years(****SD****)**		70 (9)	70 (9)	69 (9)
**Sex, female, *n* (%)**		51 (41)	27 (44)	24 (38)
**No. of comorbidities, *n* (%)**	0	30 (24)	13 (21)	17 (27)
	1	35 (28)	18 (30)	17 (27)
	2	30 (24)	12 (20)	18 (29)
	3	17 (14)	12 (20)	5 (8)
	4	9 (7)	5 (8)	4 (6)
	5	3 (2)	1 (1)	2 (3)
**BMI (****SD****)**		26 (6)	26 (7)	26 (4)
**ASA physical classification score, *n* (%)**	1	13 (10)	7 (11)	6 (10)
	2	83 (66)	37 (61)	46 (73)
	3	28 (23)	17 (28)	11 (17)
**Surgical category, *n* (%)**				
Upper gastrointestinal		11 (9)	7(11)	4 (6)
Colorectal		90 (73)	38 (62)	52(83)
Pancreatic		14 (11)	10 (16)	4(6)
Hepato-biliary		8 (6)	6 (10)	2(3)
Breast		1 (1)	0 (0)	1(2)
**Length of surgery, h (IQR)**		5 (3–8)	5 (3–8)	5 (3–7)
**Hospital stay, day (IQR)**		10 (7–16)	12 (8–20)	9 (7–14)
**Deceased 30 days, *n* (%)**		1 (1)	1 (1)	0 (0)
**Deceased 1 year, *n* (%)**		10 (8)	7 (11)	3 (5)
**QoR-15 data**				
Baseline, m (IQR), *n*=123		132 (117–143)	134 (115–143)	128 (117–143)
7–10 days postoperative, m (IQR), *n*=115		118 (102–133)	106 (91–128)	122 (107–138)
30 days postoperative, m (IQR), *n*=116		127 (115–140)	125 (103–135)	130 (119–141)
1 yr postoperative, m (IQR), *n*=92		139 (125–145)	139 (121–144)	139 (127–145)
LoR, baseline to 7–10 days, *n* (%), *n*=114		66 (58)	36 (68)	30 (49)
LoR, baseline to 30 days, *n* (%), *n*=116		53 (46)	33 (59)	20 (33)
LoR, baseline to 1 year, *n* (%), *n*=92		22 (24)	13 (33)	9 (17)

Complications registered in the study included perioperative myocardial injury (PMI); major adverse cardiac and cerebrovascular events defined as acute myocardial infarction, heart failure, new arrhythmia, nonfatal cardiac arrest, angina, pulmonary oedema, and stroke; delirium; infections (superficial and deep wound infections, bloodstream infections, urinary tract infection, pneumonia); pulmonary embolism; anastomotic leakage; postoperative bleeding; and acute kidney injury.

### Risk factors for lack of recovery

The number of events (lack of recovery) was 53 at 30 days and 22 at 1 year, and four variables were included in the multivariable models. Among the risk factors examined, only postoperative complications at 30 days were associated with lack of recovery. After adjustment for ASA class, age, and sex, complications were associated with a two-fold increased odds of lack of recovery at 30 days. No associations were observed at 1 yr (Table 2). As an MCID of 6 points may not capture nuances, a result-driven *post hoc* explorative ancillary analysis using delta QoR-15 (change from baseline) as the outcome supported the main findings, demonstrating that postoperative complications were associated with lower scores at 30 days (−7.82; 95% confidence interval [CI] −15.01 to −0.63; *P*=0.011) (Supplementary Table 2).

**Table 2:** Multivariable logistic regression analyses between risk factors and lack of recovery at 30 days and 1 yr. aOR, adjusted odds ratio; CI, confidence interval; PS, Physical Status.

Multivariable logistic regression	Lack of postoperative recovery at 30 days (*n*=53)		Lack of postoperative recovery at 1 yr (*n*=22)	
	aOR (95% CI)	*P*-value	aOR (95% CI)	*P*-value
**Complication**	2.60 (1.18–5.73)	0.018	1.76 (0.66–4.75)	0.261
**Age**	1.04 (0.99–1.09)	0.106	1.03 (0.97–1.09)	0.293
**ASA PS class**	-	0.242	-	0.871
1 (reference)	-	-	-	-
2	.83 (0.23–2.95)	0.775	1.08 (0.19–6.02)	0.929
3	.36 (0.08–1.68)	0.195	1.47 (0.21–10.56)	0.700
**Sex (reference: female)**	1.82 (0.80–4.13)	0.153	1.34 (0.46–3.92)	0.599

Within 30 days after surgery, 61 patients (49%) had at least one complication. Among these, 20 patients (16%) developed two or more complications. The most common complications were infections (41%), anastomotic leak (7%), delirium (8%), and acute kidney injury (8%). A total of 15 patients experienced PMI, and nine of them developed a postoperative complication and were included in the complication group (Supplementary Table 3). When stratified by number of complications, a gradient effect was observed. Patients experiencing two or more complications had a higher likelihood of lack of recovery compared with those with no or a single complication (odds ratio 6.48; 95% CI 1.91–21.97; *P*=0.003).

### The course of recovery

**Fig 2:**
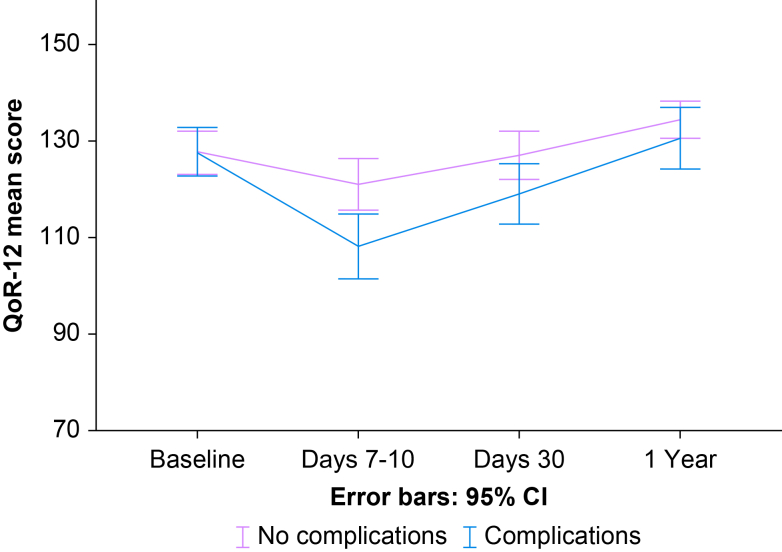
Illustration of the course of recovery over time in patients with and without postoperative complications derived from linear mixed-effects models, with error bars indicating 95% CIs. CI, confidence interval; QoR-15, Quality of Recovery-15.

A linear mixed-model analysis showed a significant change in QoR-15 scores over 1 year [*F*(3337.3)=25.17; *P*<0.001]. Compared with baseline, scores were lower at 7–10 days (−22.4; 95% CI −28.7 to −16.0) and at 30 days (−11.5; 95% CI −17.8 to −5.3), exceeding the MCID. By 1 year, scores had returned to near baseline levels. Postoperative complications influenced the course of recovery, as demonstrated by a significant interaction between time and complications [*F*(1126.9)=4.37; *P*=0.039]. Patients with complications had lower scores, with a greater decline from baseline than those without complications (Fig. 2).

The greatest difference between groups occurred at postoperative days 7–10, with lower QoR-15 scores in patients with complications (108.4±24.5) than in those without (120.8±21.0), a mean difference of 12.4 points (95% CI 3.2–20.3). The difference persisted at 30 days but was less pronounced (7.5 points; 95% CI 0.6–15.8). By 1 year, scores had converged (130.8±20.2 *vs* 133.6±13.2), with a non-significant mean difference of 2.8 points (95% CI −2.9 to 11.0).

**Fig 3:**
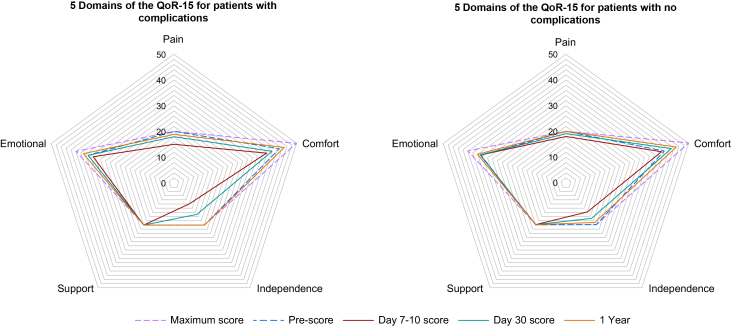
Illustration of each domain of the QoR-15 for patients with complications and without complications. Maximum possible scores for each domain were physical comfort (50), physical independence (20), emotional state (40), psychological support (20), and pain (20). QoR-15, Quality of Recovery-15.

All QoR-15 domains followed a similar pattern over 1 year, regardless of complications. At baseline, physical comfort and emotional state had the lowest scores. At postoperative days 7–10, pain and physical independence declined most. At 30 days, all domains improved and 1-year scores exceeded baseline in almost all areas. Pain continued to be notably affected in patients with complications, whereas psychological support stayed stable throughout the recovery period (Fig. 3).

To explore item-level differences, an ancillary analysis of the QoR-15 was conducted, revealing differences between patients with and without postoperative complications for items related to pain, sleep quality, personal hygiene, and usual activities (Supplementary Table 4).

### Days alive and out of hospital

The multivariable linear regression analysis, with DAOH as the dependent variable and lack of recovery at 7–10 days after surgery as the independent variable, showed that patients reporting lack of recovery had 3.7 fewer DAOH at 30 days after surgery (mean difference −3.69 [−6.54 to −0.83]; *P*=0.012). The association remained significant after adjustment for age, ASA class, and sex (Table 3).

**Table 3:** Multivariable linear regression analysis of associations between lack of recovery and DAOH at 30 days. CI, confidence interval; DAOH, days alive and out of hospital.

Multivariable linear regression	DAOH at postoperative day 30 (*n*=124)	
	Adjusted mean difference in DAOH (days) (95% CI)	*P*-value
**Lack of recovery, days 7–10**	−3.69 (−6.54 to −0.83)	0.012
**Age**	−0.05 (−0.21 to 0.11)	0.535
**ASA Physical Classification Score**	−0.84 (−3.43 to 1.76)	0.524
**Sex**	0.40 (−2.59 to 3.39)	0.791

We found that most early dropouts were caused by limited availability of study nurses. Of the nine patients lost at the 7–10-day follow-up, seven were available at 30 days. At 1 yr, 92 patients remained, and among those lost, 10 had died, and 22 did not respond. The median 30-day QoR-15 score was 127 (IQR 115–140) in the full cohort and 125.5 (IQR 120–143) among 1-year non-responders. These patients did not differ from the overall cohort in age, ASA class, or sex but had a higher rate of postoperative complications (63% *vs* 49%).

## Discussion

Our findings demonstrate that lack of recovery is common after major abdominal surgery, with the highest incidence observed at 7–10 days after surgery. Although some degree of recovery impairment in the early postoperative period is expected,^10^^,^^11^ it is notable that 58% of patients reported being worse off at this time point. Although this declines over 30 days and further over 1 year, a considerable proportion of patients continue to report a lack of recovery. One in five patients reported lack of recovery at 1 year, which highlights a long-term impact of major surgical procedures.

Patients who developed postoperative complications had two-fold higher odds of reporting lack of recovery at 30 days. In addition, early lack of recovery was independently associated with fewer DAOH, indicating that early PROs may serve as indicators of healthcare burden within the first 30 postoperative days.

Our results showing that a substantial proportion of patients experience incomplete recovery align with previous studies demonstrating persistent symptoms and reduced quality of life months after surgery, underscoring the need for prolonged follow-up and targeted postoperative support,^10^^,^^18^^,^^25^ and suggesting that QoR-15 may be a sensitive and useful measure in long-term follow-up.

This finding underscores the extent and burden of lack of recovery after major surgery in a patient group known to be at high risk of altered recovery.^12^ Although this cohort represented a low-to-intermediate risk population, with 76% of patients classified as ASA I–II, the observed decline suggests clinically relevant deterioration that warrants attention. Most patients (73%) underwent colorectal surgery. The mean age was 70 years, and nearly half of the patients had multiple comorbidities, indicating limited physiological reserve and increased vulnerability. A median hospital stay of 10 days further reflects the complexity of the perioperative course.

Few studies have evaluated short- and long-term lack of recovery after major, particularly abdominal surgery, and no consensus exists on its definition.^26^ We evaluated recovery relative to each patient’s baseline QoR-15 score, allowing detection of meaningful individual changes over time without relying on absolute or categorical recovery measures. We defined lack of recovery as a ≥6-point decrease in QoR-15 score compared with baseline. This threshold was chosen based on previous studies indicating that the change corresponds to the MCID.^9^ From a clinical perspective, this change is meaningful for patients and provides a pragmatic definition of lack of recovery. However, its relevance for long-term recovery requires careful consideration. QoR-15 was originally developed for early postoperative recovery, and longer follow-up may reflect broader aspects of general health status rather than surgery-specific recovery. Therefore, the long-term findings should be interpreted in this context.

The finding that postoperative complications emerged as a risk factor associated with lack of recovery is not surprising and consistent with earlier research linking postoperative complications to delayed recovery^19^ after both emergency^25^ and day surgery.^27^ However, postoperative complications were the only identified risk factor for lack of recovery that may be modifiable. This indicates that the initial postoperative days may be a critical period, as early events can be symptoms of further deterioration^28^ and a large part of postoperative complications occur during this phase.^29^ This emphasises the need for high-quality perioperative care to prevent, detect, and manage complications. Thorough planning of postoperative care can reduce unplanned intensive care and hospital days,^30^ whereas enhanced recovery guidelines emphasise coordinated interventions throughout the perioperative process.^31^

Infections accounted for most 30-day complications, followed by delirium, acute kidney injury, and anastomotic leakage. Crucially, half of the study participants experienced at least one complication within 30 days after surgery. This is partly because 73% of participants underwent colorectal surgery, consistent with previous literature reporting complication rates up to 66% in this surgical population.^32^ Given that age, ASA class, and sex were not associated with lack of recovery, the outcome is unlikely to be explained solely by baseline vulnerability. Rather, potentially preventable complications seem to have a central role and are more directly linked to the development of lack of recovery.

Exploratory analyses of QoR-15 domains provided a more in-depth analysis of recovery, with similar temporal patterns across domains regardless of complications. At baseline, the lowest scores were seen in ‘physical comfort’ and ‘emotional state’, indicating that preoperative anxiety and uncertainty might be present and perhaps preventable through information and additional support. By contrast, at 7–10 days after surgery, pain and physical independence were the most important contributors to lower total scores. Low scores in the domain of physical independence may be partly procedure specific and related to adaptation to a temporary colorectal stoma, which several patients in our study described as a burden. At 30 days, improvements were seen in all domains, and at 1 year, scores exceeded baseline in almost all domains. In current literature, severe pain on postoperative day 4 has been linked to poor recovery at 1 week and 1 month and persisted up to 1 year in 25% of patients.^33^ Inadequate pain management may be a key determinant of the course of recovery, as uncontrolled pain can impair mobility, disrupt sleep, and negatively influence mood. This underscores the need for early pain assessment and management to improve comfort and potentially support long-term recovery.

To further explore recovery, we assessed the course over a 1-year period. Patients with complications demonstrated a steeper early decline in QoR-15 scores and a slower recovery than those without complications. Although differences persisted at 30 days, they were less pronounced, suggesting partial recovery over time. Most QoR-15 studies focus on early time points,^34^ whereas long-term recovery trajectories are less well described. Our findings extend existing literature by showing that postoperative complications are associated with sustained impairment in patient-perceived recovery over time, but do not fully explain long-term outcomes. Notably, two of five patients who had not recovered in 1 year had no complications, suggesting that factors beyond complications contribute to persistent lack of recovery. Cancer- and treatment-related effects, chronic symptoms, psychological distress, comorbidity, and reduced functional reserve reasonably play a role, although this cannot be determined from present data.

Early lack of recovery at 7–10 days after surgery was associated with a substantial reduction in DAOH at 30 days, consistent with QoR-15 validation in emergency surgery.^28^ In turn, fewer DAOH was associated with higher morbidity and 1-year mortality in several studies.^21^^,^^22^^,^^35^ Linking these measures is valuable, as an early lack of recovery may signal adverse outcomes before complications arise, whereas DAOH can guide the targeted use of QoR-15. However, DAOH is also influenced by contextual factors such as discharge policies and care organisation. Our result is in line with a French validation study of QoR-15 in emergency surgery that showed early recovery correlated with long-term PROMs such as quality of life and number of days at home after surgery.^25^

Several limitations merit consideration. First, the small sample size resulted in wide CIs and limited precision. Although the direction of associations was consistent, magnitude should be interpreted with caution and confirmed in larger studies. *Post hoc* power analysis suggested adequate power at 30 days, but likely underpowered at 1 year, owing to the low number of events. The limited number of events (*n*=53 at 30 days and *n*=22 at 1 year) also restricted the number of variables in the multivariable model. Other relevant factors, such as oncological treatments, surgical technique, and intraoperative variables, were unavailable and may have influenced the result.

Second, lack of recovery was defined as a ≥6-point decrease in QoR-15 based on established MCID. This threshold may be influenced by measurement variability, and dichotomisation may have introduced some misclassification. This is particularly relevant at longer-term follow-up, where the MCID was originally established in early postoperative settings, and its applicability is less certain. To address this, we analysed the continuous change in QoR-15, which showed a similar pattern, supporting the robustness of the findings.

Third, survivor bias should be considered as patients with poorer recovery or more severe complications may have been less likely to complete later follow-up. This may lead to an overestimation of late recovery and an underrepresentation of persistent impairment. However, individuals lost to follow-up did not differ in baseline characteristics or QoR-15 scores, which reduces but does not eliminate the potential for bias.

QoR-15 is straightforward to administer, but optimal timing and establishing clear criteria for interpreting scores are challenging.^26^ Given the absence of consensus on defining lack of recovery, future research may benefit from focusing on the course of recovery rather than single time points and addressing patient- and treatment-related factors across the first postoperative year.

Generalisability may be limited by the convenience sample, specific inclusion criteria, and the predominance of colorectal surgery in a Swedish perioperative context, potentially restricting applicability to broader surgical populations and other healthcare systems. However, the use of a validated PROM (QoR-15) and longitudinal follow-up supports relevance to similar patient groups.

## Conclusion

We report that more than half of the patients reported lack-of-recovery scores at 7–10 days and that this pattern persisted even at 1 month after surgery. Postoperative complications within 30 days were common and strongly associated with lack of recovery after surgery, regardless of patients’ baseline health status. Moreover, early lack of recovery was linked to a significant reduction in days alive out of hospital, suggesting that early patient reported outcomes may provide useful signals of later healthcare needs, further underscoring the need for larger, multicentre studies before adopting routine changes to clinical pathways.

## Authors’ contributions

Had full access to all data in the study and take full responsibility for the integrity of the data and the accuracy of the data analysis: SL, MSC, JS

Concept and design: SL, MSC

Acquisition: SL, MSC, AN, HD, CJ, HA, JS

Analysis and data interpretation: SL, MSC, JS

Drafting of the manuscript: SL, MSC, JS

Clinical revision of manuscript for important intellectual content: all authors

Obtained funding: SL, MSC

## Funding

Funding from Centre for Clinical Research Sörmland, Uppsala University, Eskilstuna, Sweden to SL. Funding from the Swedish Research Council (2019-02833), South Eastern Sweden Research Council (746981, 712291), and Linköping University-Region Östergötland ALF (687681, 792291) to MSC.

## Declarations of interest

MSC has received honoraria/speaker’s fees from AOP Health, Becton Dickinson, Philips Healthcare, Laboratorie Agguetant, and Getinge. All other authors declare that they have no conflicts of interest.
