# Supplementary material for: Incidence and risk factors for lack of recovery in patients undergoing major abdominal noncardiac surgery in Sweden: a prospective observational study
Source: BJA Open. 2026 Jul 29;19:100645. doi: 10.1016/j.bjao.2026.100645 (PMC13451793; doi:10.1016/j.bjao.2026.100645)
Supplement: Supplementary file 1 [file mmc1.docx]

**Supplementary material**

**Table of contents	Page**

| **Supplementary Table S1.** | STROBE reporting checklist. | **2** |
| --- | --- | --- |
| **Supplementary Table S2.** | Three separate multivariable linear regression analyses between risk-factors and the QoR-15 delta score from baseline. | **4** |
| **Supplementary Table S3.** | The 30-day incidence of postoperative complications. | **5** |
| **Supplementary Table S4.** | Item-level analysis of the QoR-15 scores at 7-10 days postoperatively. | **6** |

**Supplementary Table S1.** STROBE Statement—checklist of items that should be included in reports of observational studies

|  | Item No. | Recommendation | Page  No. | | |  |  |  |
| --- | --- | --- | --- | --- | --- | --- | --- | --- |
| **Title and abstract** | 1 | (*a*) Indicate the study’s design with a commonly used term in the title or the abstract | 1 | | |  |  |  |
|  |  | (*b*) Provide in the abstract an informative and balanced summary of what was done and what was found | 2 | | |  |  |  |
| Introduction | | | | | |  |  |  |
| Background/rationale | 2 | Explain the scientific background and rationale for the investigation being reported | 4-5 | | |  |  |  |
| Objectives | 3 | State specific objectives, including any prespecified hypotheses | 7 | | |  |  |  |
| Methods | | | | | |  |  |  |
| Study design | 4 | Present key elements of study design early in the paper | 5 | | |  |  |  |
| Setting | 5 | Describe the setting, locations, and relevant dates, including periods of recruitment, exposure, follow-up, and data collection | 5-6 | | |  |  |  |
| Participants | 6 | (*a*) *Cohort study*—Give the eligibility criteria, and the sources and methods of selection of participants. Describe methods of follow-up  *Case-control study*—Give the eligibility criteria, and the sources and methods of case ascertainment and control selection. Give the rationale for the choice of cases and controls  *Cross-sectional study*—Give the eligibility criteria, and the sources and methods of selection of participants | 5-6 | | |  |  |  |
|  |  | (*b*) *Cohort study*—For matched studies, give matching criteria and number of exposed and unexposed  *Case-control study*—For matched studies, give matching criteria and the number of controls per case | - | | |  |  |  |
| Variables | 7 | Clearly define all outcomes, exposures, predictors, potential confounders, and effect modifiers. Give diagnostic criteria, if applicable | 6-7 | | |  |  |  |
| Data sources/ measurement | 8* | For each variable of interest, give sources of data and details of methods of assessment (measurement). Describe comparability of assessment methods if there is more than one group | 7 | | |  |  |  |
| Bias | 9 | Describe any efforts to address potential sources of bias | 8 | | |  |  |  |
| Study size | 10 | Explain how the study size was arrived at | 7 | | |  |  |  |
| Quantitative variables | 11 | Explain how quantitative variables were handled in the analyses. If applicable, describe which groupings were chosen and why | | 7-8 |  | | |  |
| Statistical methods | 12 | (*a*) Describe all statistical methods, including those used to control for confounding | | 7-8 |  | | |  |
|  |  | (*b*) Describe any methods used to examine subgroups and interactions | | 7-8 |  | | |  |
|  |  | (*c*) Explain how missing data were addressed | | 8 |  | | |  |
|  |  | (*d*) *Cohort study*—If applicable, explain how loss to follow-up was addressed  *Case-control study*—If applicable, explain how matching of cases and controls was addressed  *Cross-sectional study*—If applicable, describe analytical methods taking account of sampling strategy | | 6, 11 |  | | |  |
|  |  | (*e*) Describe any sensitivity analyses | | 9 |  | | |  |
| Results | | | | | | | |  |
| Participants | 13* | (a) Report numbers of individuals at each stage of study—eg numbers potentially eligible, examined for eligibility, confirmed eligible, included in the study, completing follow-up, and analysed | | 8 |  | | |  |
|  |  | (b) Give reasons for non-participation at each stage | | 8 |  | | |  |
|  |  | (c) Consider use of a flow diagram | | 8 |  | | |  |
| Descriptive data | 14* | (a) Give characteristics of study participants (eg demographic, clinical, social) and information on exposures and potential confounders | | 8 |  | | |  |
|  |  | (b) Indicate number of participants with missing data for each variable of interest | | 7 |  | | |  |
|  |  | (c) *Cohort study*—Summarise follow-up time (eg, average and total amount) | | 8 |  | | |  |
| Outcome data | 15* | *Cohort study*—Report numbers of outcome events or summary measures over time | | 8 |  | | |  |
|  |  | *Case-control study—*Report numbers in each exposure category, or summary measures of exposure | | *-* |  | | |  |
|  |  | *Cross-sectional study—*Report numbers of outcome events or summary measures | | - |  | | |  |
| Main results | 16 | (*a*) Give unadjusted estimates and, if applicable, confounder-adjusted estimates and their precision (eg, 95% confidence interval). Make clear which confounders were adjusted for and why they were included | | 8 |  | | |  |
|  |  | (*b*) Report category boundaries when continuous variables were categorized | | - |  | | |  |
|  |  | (*c*) If relevant, consider translating estimates of relative risk into absolute risk for a meaningful time period | | - |  | | |  |
| Other analyses | 17 | Report other analyses done—eg analyses of subgroups and interactions, and sensitivity analyses | | - | |  | | |
| Discussion | | | | | | | | |
| Key results | 18 | Summarise key results with reference to study objectives | | 11 | |  | | |
| Limitations | 19 | Discuss limitations of the study, taking into account sources of potential bias or imprecision. Discuss both direction and magnitude of any potential bias | | 15 | |  | | |
| Interpretation | 20 | Give a cautious overall interpretation of results considering objectives, limitations, multiplicity of analyses, results from similar studies, and other relevant evidence | | 11-15 | |  | | |
| Generalisability | 21 | Discuss the generalisability (external validity) of the study results | | 16 | |  | | |
| Other information | |  | | | | | | |
| Funding | 22 | Give the source of funding and the role of the funders for the present study and, if applicable, for the original study on which the present article is based | | 20 | |  | | |

*Give information separately for cases and controls in case-control studies and, if applicable, for exposed and unexposed groups in cohort and cross-sectional studies. **Note:** An Explanation and Elaboration article discusses each checklist item and gives methodological background and published examples of transparent reporting. The STROBE checklist is best used in conjunction with this article (freely available on the Web sites of PLoS Medicine at <http://www.plosmedicine.org>

/, Annals of Internal Medicine at <http://www.annals.org/>, and Epidemiology at http://www.epidem.com/). Information on the STROBE Initiative is available at www.strobe-statement.org.

**Supplementary Table S2.** Three separate multivariable linear regression analyses between risk-factors and the QoR-15 delta score from baseline

| **Multivariable linear regression** | **Delta QoR15 from preoperative baseline and**  **7-10 days** | | **Delta QoR15 from preoperative baseline and 30 days** | | **Delta QoR15 from preoperative baseline and 1year** | |
| --- | --- | --- | --- | --- | --- | --- |
|  | **β coefficient (95% CI)** | **P value** | **β coefficient (95% CI)** | **P value** | **β coefficient (95% CI)** | **P value** |
| **Complication** | -11.72 (-20.67- -2.77) | .011 | -7.82 (-15.01- -.63) | .033 | -3.94(-10.56-2.68) | .240 |
| **Age** | -.55 (-1.05- -.04) | .035 | -.34 (-.75-.07) | .107 | -.32 (-6.99-.08) | .104 |
| **ASA PS- class** | 9.69 (1.22-18.15) | .025 | 5.09 (-1.62-11.79) | .136 | 1.07(-5.26-7.40) | .738 |
| **Sex** | -8.25 (-17.88-1.37) | .092 | -4.01 (-11.50-3.48) | .291 | -1.60(-8.62-5.43) | .653 |
| Abbreviations: n =numbers of participants, 95% CI= confidence interval, ASA-PS= American Society of Anesthesiologists Physical Status Classification. | | | | | | |

**Supplementary Table S3.** The 30-day incidence of postoperative complications.

| **30-day postoperative complications** | **Number of patients** | **% of the study-group, (n= 124)** |
| --- | --- | --- |
| MACCE | 7 | 5.6% |
| Pulmonary embolism | 1 | 0.8% |
| Anastomotic leak | 9 | 7.3% |
| Acute kidney injury | 10 | 8.1% |
| Postoperative bleeding | 6 | 4.8% |
| Delirium | 10 | 8.1% |
| Pneumonia | 3 | 2.4% |
| Superficial SSI | 19 | 15.3% |
| Deep incisional SSI | 1 | 0.8% |
| Organ space SSI | 12 | 9.7% |
| Bloodstream infection | 8 | 6.5% |
| Urinary tract infection | 8 | 6.5% |
| Abbreviations: MACCE= Major Adverse Cardiac or Cerebrovascular Events, SSI= Surgical Site Infection, n=number | | |

**Supplementary Table S4.** Item-level analysis of the QoR-15 scores at 7-10 days postoperatively.

| **QoR-15 items, days 7-10**  **median (IQR)** | | **Domain** | **Total group (n=115)** | **With complications (n=53)** | **Without complications (n=62)** |
| --- | --- | --- | --- | --- | --- |
| *Able to breathe easily* | | Physical  Comfort | 10 (8-10) | 10 (8-10) | 10 (9-10) |
| *Been able to enjoy food* | | Physical  comfort | 6(1-9) | 5(0-8) | 7 (4-9) |
| *Feeling rested* | | Physical  comfort | 7 (5-8) | 7 (5-8) | 8 (5-9) |
| *Have had good sleep* | | Physical  comfort | 7 (5-10) | 7 (3-9) | 8 (6-10) |
| *Able to look after personal hygiene unaided* | | Physical independence | 10 (5-10) | 9 (5-10) | 10 (8-10) |
| *Able to communicate with family and friends* | | Psychological support | 10 (10-10) | 10 (10-10) | 10 (10-10) |
| *Getting support from hospital, doctors, nurses* | | Psychological support | 10 (10-10) | 10 (9-10) | 10 (10-10) |
| *Able to return to work or usual activities* | | Physical independence | 2 (0-6) | 0 (0-5) | 5 (0-7) |
| *Feeling comfortable and in control* | | Emotional  state | 9 (8-10) | 9 (7-10) | 10 (8-10) |
| *Having a feeling of general well-being* | | Emotional  state | 7 (4-9) | 7 (4-9) | 8 (5-10) |
| *Moderate pain* | | Pain | 7 (5-10) | 6 (5-10) | 8 (6-10) |
| *Severe pain* | | Pain | 10 (9-10) | 10 (7-10) | 10 (10-10) |
| *Nausea and vomiting* | | Physical  comfort | 10 (8-10) | 10 (7-10) | 10 (8-10) |
| *Feeling worried or anxious* | | Emotional  state | 10 (8-10) | 10(8-10) | 10 (8-10) |
| *Feeling sad or depressed* | | Emotional  state | 10 (8-10) | 10 (8-10) | 10 (8-10) |
| **Total score** | |  | 120 (100-138) | 106 (92-128) | 122 (106-138) |
| Abbreviations: QoR= Quality of Recovery-15 score, n =number of participants and IQR=interquartile range. | | | | | |
|  |  |  |  |  |  |
